# Supplementary material for: Anisotropy-driven quantum criticality in an intermediate valence system
Source: Nat Commun. 2022 Apr 19;13:2141. doi: 10.1038/s41467-022-29757-9 (PMC9019086; doi:10.1038/s41467-022-29757-9)
Supplement: Supplementary file 1 — Suppplementary information [file 41467_2022_29757_MOESM1_ESM.pdf]

Supplementary Information: Anisotropy-driven quantum criticality in an intermediate valence system, M. S. Grbić et al.

Supplementary Note 1

THERMAL EXPANSION IN VARIOUS YB-BASED SYSTEMS

In $\alpha-$YbAlB_4 at $B = 0$ and low temperatures the linear thermal expansion coefficient $\alpha_i = d\left(\Delta L_i/L_i\right)/dT$ is positive for both the $i = a$ and c axes. The volume thermal expansion α_{Vol}, which is dominated by the c-axis is consequently positive. This is surprising since in Yb-based KL or IV systems α_{Vol} is always negative as is the magnetostriction $\lambda_i = d\left(\Delta L_i/L_i\right)/dB$. The reason is that $\alpha_{Vol} = -V_m^{-1}(dS/dp)_{T,B}$ (S = entropy, p = pressure, V_m = molar volume) measures the pressure dependence of the entropy associated to the relevant energy scale of a system. In typical Yb-based KL systems, with the T_K as a relevant energy scale, pressure stabilizes the magnetic Yb^{3+}, which is smaller than the non-magnetic Yb^{2+}, yielding $dT_K/dp < 0$ (i.e. $(dS/dp)_{T,B} > 0$) so that $\alpha_{Vol} < 0$.

In metamagnetic systems where the entropy is dominated by magnetic fluctuations the the thermal expansion can be expressed as $\alpha_{Vol} = \Omega V_m^{-1}(\partial S/\partial B)_T$, where $\Omega = (dB_c/dp)_{B=B_c}$ is constant for small pressures and with B_c the critical field

Supplementary Note 2

NMR DATA

^{11}B NMR spectrum across the transition at B_2

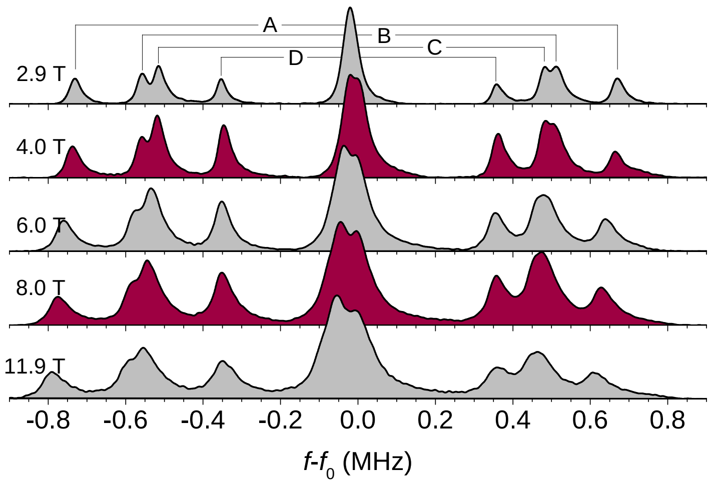

Supplementary Figure 1. ^{11}B NMR spectra for different magnetic field values, measured at 1.34 K for 2.9 T and at 3 K for 4 T, 6 T, 8 T and 11.9 T, respectively. The spectra show a central line with four pairs of quadrupolar satellites originating from four crystallographically different sites of boron in α-YbAlB$_4$. The spectra have been centred and shifted on top of each other for clarity.

We show the complete NMR spectra of ^{11}B and how it evolves with magnetic field at 1.34 K. The spectra show a central line of higher intensity with four pairs of satellites, which correspond to four crystallographically inequivalent positions of boron in α-YbAlB$_4$ as indicated by different colour coded sites in Fig. 1a). At 2.9 T all the central lines overlap, but with the increase of magnetic field they start to distinguish because their Knight shift values (K) slightly differ. This is in accordance to the previously determined conclusion of Ref. 1. For consistency, we keep the same naming of the sites (A - D) as in Ref. 1. In this paper we present the data recorded on site D, for magnetic field along the c axis. This site was chosen because it has a well separated NMR line (unlike sites B and C), which is important for T_1 measurements, and has a shorter T_1 relaxation time than site A. The ^{11}B NMR spectra show no qualitative change (Supplementary Figure 1) as the magnetic field is varied across B_2= 3.6 T. The shifts of the central lines, as well as the slowly increasing linewidths, follow the behaviour of the macroscopic magnetization with no change in the

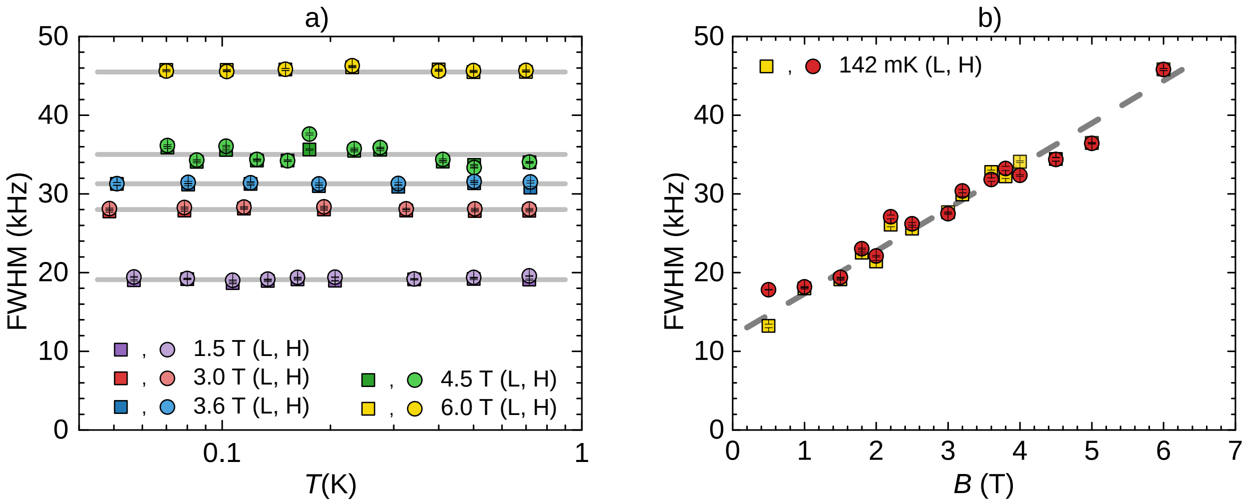

Supplementary Figure 2. a) Temperature dependence of FWHM values of the ^{11}B site D, measured at several magnetic field values. The values determined for the low frequency satellite (L) are marked by squares, while those for the high frequency satellite (H) are marked by circles. The grey full lines are only guides for the eye that show the temperature independence of the measured values. b) Magnetic field dependence of FWHM values of ^{11}B site D, measured at 142 mK. The grey dashed line is only a guide for the eye that shows the linear magnetic field dependence. Vertical error bars in the figures are least-square fit errors (1 s.d.) of fitting NMR lines to a Gaussian.

shape of the spectrum. The behaviour of the boron D site's linewidths were measured with temperature and magnetic field down to very low temperatures and shown as half width at half maximum (FWHM) values in Supplementary Figure 2. It is visible from the data that FWHM values are temperature independent down to lowest values, and that it linearly increases with magnetic field. Supplementary Figure 2 shows FWHM values for both satellite lines, the lower frequency one (L) and the higher (H) frequency one. The lack of abrupt changes in FWHM and no temperature dependence rule out magnetic or charge order appearing at B_1 or B_2 that would break the translational symmetry and considerably modify the NMR spectra. The linear increase of FWHM with magnetic field indicates a (small, but present) distribution of local susceptibility values (K), that originates from small, but unavoidable inhomogeneity of the sample. However, FWHM value of 50 kHz is still quite small and indicates that the sample is of high quality. We additionally confirm the lack of magnetic or charge order by showing the complete behaviour of the Knight shift data in the next section.

Knight shift data of ^{11}B and ^{27}Al

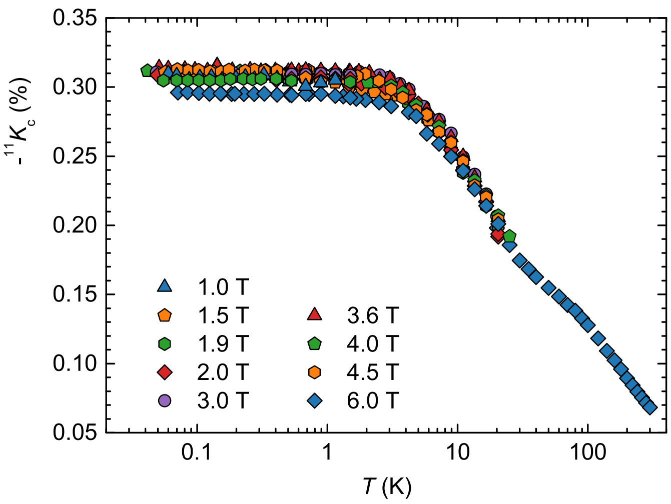

Supplementary Figure 3. ^{11}B Knight shift vs. temperature at different values of magnetic field. Vertical error bars in the figures are smaller or equal to symbol size and originate from a least-square fit errors (1 s.d.) of fitting NMR lines to a Gaussian.

We recorded the Knight shift of boron D site (^{11}K) in a very broad temperature range from 300 K to 50 mK. In the temperature region from 300 K to 10 K the values of c-axis ^{11}K rapidly change since there the bands hybridize (the valence fluctuation scale is ≈ 150 K), and the system crosses from a localized-moment one to a Fermi-liquid one. As the temperature is lowered below $T^{*}\approx$ 8 K, ^{11}K values saturate to a constant value that shows only a small change with magnetic field (Supplementary Figure3). A magnetic field dependence of ^{11}K at low temperature is shown in Fig. 1 d of the main text. This virtually flat behaviour below 1 K and 4.5 T is in stark contrast to the $(T_1T)^{-1}$ data that show divergences appearing at B_1 and B_2.

Similar to the behaviour of $^{11}K_c$, the c-axis Knight shift of ^{27}Al nuclei ($^{27}K_c$) shows an equivalent temperature and magnetic field dependence (Supplementary Figure 4 a) and b)), with only a weak feature at B_2, which further confirms the absence of long-range magnetism at B_1 and B_2. Only when the magnetic field increases above 4 T, there is a gradual change in $^{27}K_c$ arising from the slow suppression of the density of states at the Fermi level $N(\epsilon_F)$ (consistent with specific heat data and susceptibility).

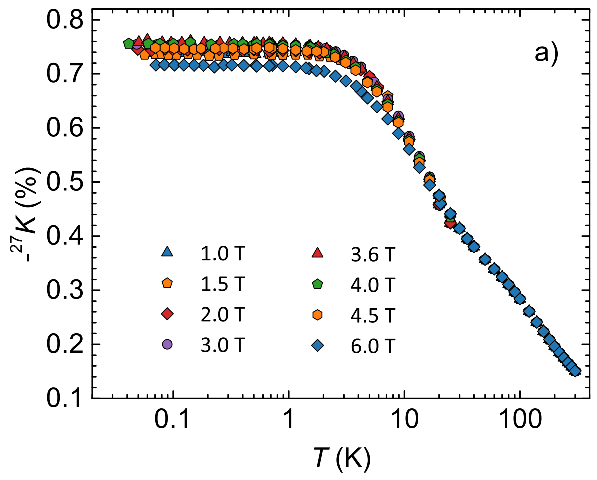

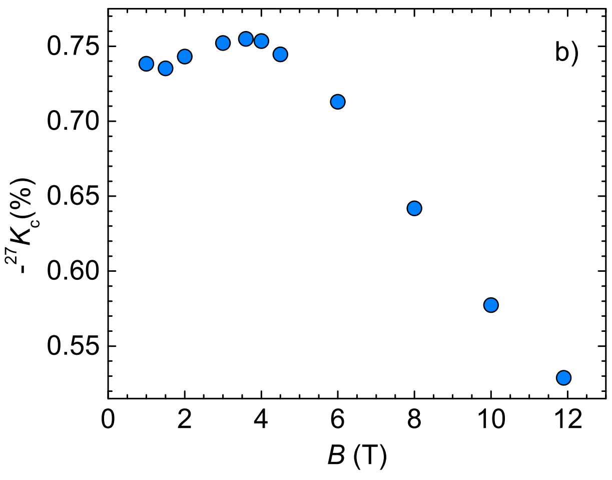

Supplementary Figure 4. a) ^{27}Al Knight shift vs. temperature at different values of magnetic field, b) ^{27}Al Knight shift vs. magnetic field, measured at 500 mK. Vertical error bars in the figures are smaller or equal to symbol size and originate from a least-square fit errors (1 s.d.) of fitting NMR lines to a Gaussian.

^{27}Al and ^{11}B quadrupolar coupling

An additional clue as to the nature of the non-Fermi liquid (NFL) behaviour at B_2 comes from the c-axis component of the quadrupolar coupling of ^{27}Al ($^{27}\nu_Q$), which first increases with field (similar to the magnetostriction λ_c) and then abruptly decreases by about 0.2% (Figure 5a)) when crossing B_2.

Two features are visible in the magnetic field dependence of $^{11}\nu_Q$, and they coincide with B_1 and B_2. Interestingly, the drop in $^{11}\nu_Q$ at B_1 is not detected by $^{27}\nu_Q$. This is consistent with the interpretation of B_1 as being a Lifshitz transition caused by anisotropic f-c hybridization, where conduction bands come mainly from B orbitals.

From the magnetic field behaviour of $^{11,27}\nu_Q$ at B_2 one might consider the possibility that the transition is a magnetic-field induced valence change, however such a transition would have a large and characteristic effect on thermodynamic properties, which are not observed. The change in valence corresponding would be detected by an X-ray absorption measurement and cause a strong signature in magnetostriction (Ref. 2). However, no change in valence has been reported[3], and magnetostriction only shows a smooth change in contrast to previously studied valence-transition materials. Even if the valence transition occurs at lower temperatures, in this case it is expected to see a broad magnetic field-induced

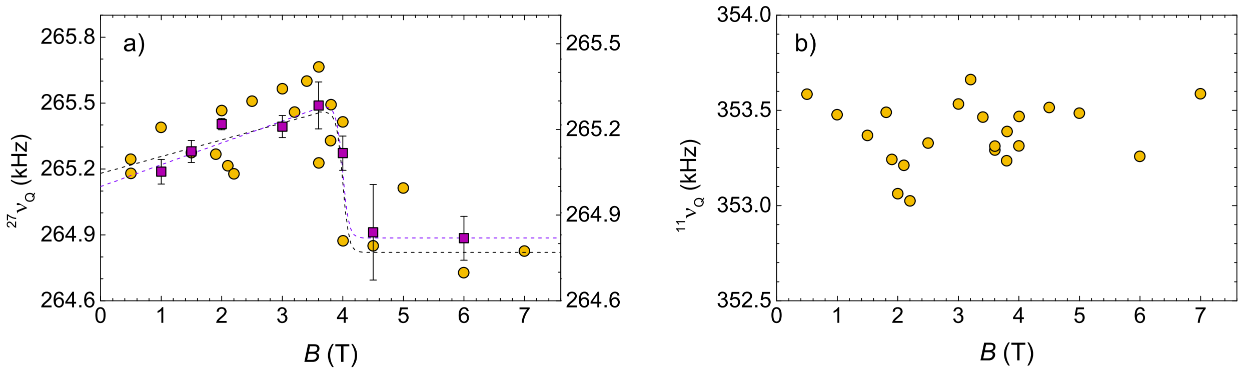

Supplementary Figure 5. a) Magnetic field dependence of the principal value of the quadrupolar splitting ν_Q of ^{27}Al ($^{27}\nu_Q$) measured at 142 mK. Yellow circles (left scale) are determined from a single measurement of the outermost satellites of the ^{27}Al NMR spectrum, while purple squares (right scale) show averaged values determined from a temperature-dependent measurement at a particular magnetic field (not shown). Namely, below 4 K $^{27}\nu_Q$ saturates to a constant value, which is why averaging can be made to lower the uncertainty of the measurement. The error bars (1 s.d.) depict the data scatter. These two data sets slightly differ in absolute value due to a small $\sim 1^\circ$ misalignment of the magnetic field with respect to the crystal c-axis for the purple squares. Dashed lines are only guides for the eye. b) Magnetic field dependence of the quadrupolar splitting of boron ($^{11}\nu_Q$) measured at 142 mK. Shown values of $^{11}\nu_Q$ were measured at the boron site with the smallest splitting (innermost lines in Supplementary Figure 1), but other sites show similar behaviour.

response detectable at relatively high temperatures. For instance, in a study[4] on $YbAgCu_4$, XAS technique observed a finite broad magnetic field-induced change in valence already at 50-55 K, even though the valence change onsets at $T_V \approx 42$ K. Similarly, in a HAXPES measurement[5] on Fe-doped α-$YbAlB_4$, a first order valence transition was detectable already at 20 K, even though the transition occurs at $T = 0$ K. Therefore, the anomaly at B_2 cannot be due to a valence transition.

A subtle structural transition can also be ruled out because it would have been observed by $^{11,27}\nu_Q$ measurements as a sharper feature, and it would also be seen clearly in magnetostriction data, which is not the case.

Recently[6] Mössbauer spectroscopy found a magnetic field induced change in the quadrupolar moment of Yb ion between 1 and 5 T. This result indicates that the phenomenon observed

at B_2 can originate from a multipole-type QCP in α-YbAlB$_4$. However, since the symmetry of the α-YbAlB$_4$ compound (and the Yb site) is already low, it will require additional work to further predict the symmetry change across B_2.

Nuclear magnetic spin-lattice relaxation rate

The NMR spin-lattice relaxation rate is related to the imaginary part of the dynamical susceptibility $\chi''(\mathbf{q}, \omega)$ as

$$(T_1T)^{-1} = \frac{\gamma_m^2 k_B}{2\mu_B^2} \lim_{\omega \to 0} \sum_q |A(\mathbf{q})|^2 \frac{\chi''(\mathbf{q}, \omega)}{\omega} \tag{1}$$

where k_B is the Boltzmann constant, μ_B is the Bohr magneton, γ_m is the gyromagnetic ratio and $A(\mathbf{q})$ is the hyperfine coupling constant. As we can see, the summation is over entire q space which makes $(T_1T)^{-1}$ sensitive to the excitations that originate also at $q > 0$, unlike the Knight shift K which is sensitive only to $q = 0$ part of the susceptibility.

For the ^{11}B measurements, we recorded the relaxation of nuclear magnetization $M(t)$ by using a saturation-pulse sequence, as described in Methods. Each measurement set contained more than 20 measurement points, and the data were fit to the recovery function $M(t) = M_0(1-(1/10)e^{-(t/T_1)^\beta}-(5/10)e^{-(3t/T_1)^\beta}-(4/10)e^{-(6t/T_1)^\beta})$, suitable for a spin $I = 3/2$ satellite transition, where β is the so-called stretch exponent. In figure 6 we show several measurements and fitted curves taken at 142 mK. In figure 7 we show the values of the β

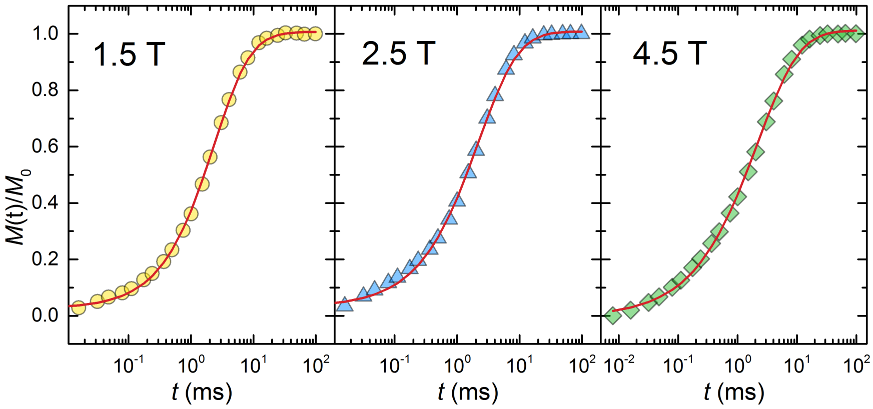

Supplementary Figure 6. Spin-lattice relaxation time measurements (symbols) of ^{11}B D site and fitted curves (red lines) determined at 1.5 T (yellow circles), 2.5 T (blue triangles) and 4.5 T (green diamonds). All data were determined at 142 mK.

parameter determined at 142 mK for the T_1 values shown in the upper paned of Fig. 2a). The β decreases gradually with field, indicating a growing inhomogeneity in the dynamical susceptibility with magnetic field.

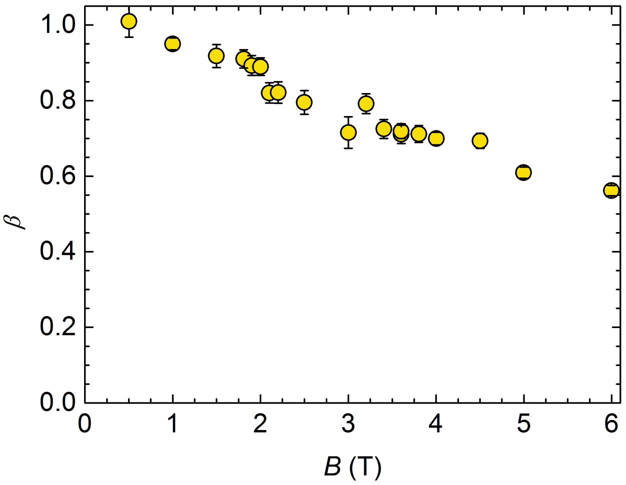

Supplementary Figure 7. Stretch parameter β determined from spin-lattice relaxation time T_1 measurements at various magnetic fields and at 142 mK. Vertical error bars in the figures originate from a least-square fit errors (1 s.d.) of fitting to a relaxation curve defined in the text.

Supplementary Note 3

RESISTIVITY DATA

Exponent of the resistivity within the $ab-$plane

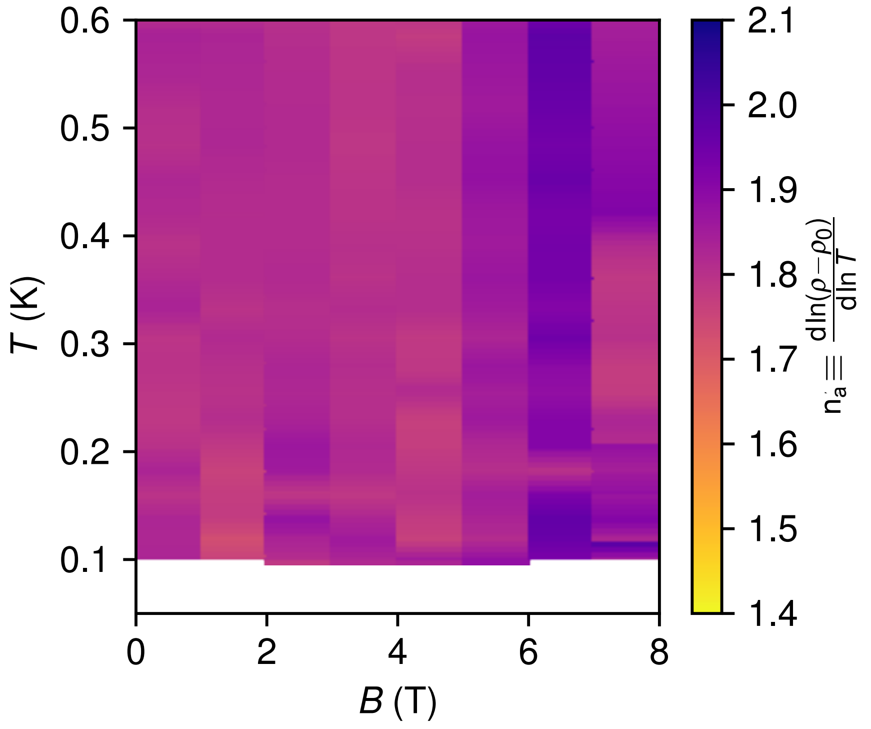

Supplementary Figure 8. Resistivity exponent with current $I \parallel a$ and $B \parallel c$, displayed on the same colour scale as in the main text.

Figure 8 shows the n_a exponent of the resistivity as defined in the main text for current applied parallel to the a-axis. Consistent with[7] we observe $n_a \approx 1.8$ at $B = 0$. n_a is approximately temperature and magnetic field independent indicating the strong anisotropy of critical fluctuations, which are primarily confined to the c-axis.

Spin zeros

When a quasi 2D Fermi surface (FS) is spin split by the Zeeman interaction the Shubnikov-de Haas oscillations of spin up and down FS interfere leading to an amplitude

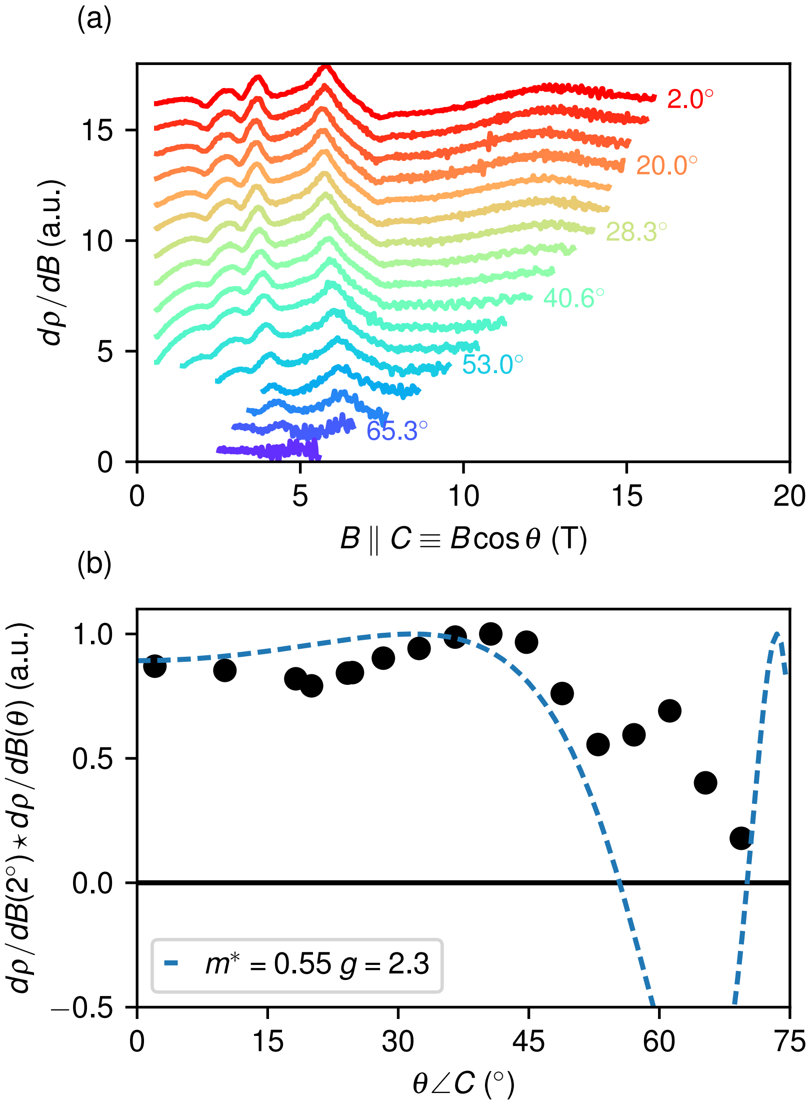

Supplementary Figure 9. (a) $d\rho/dB$ against $B \parallel c$ at various angles of B in the [001] → [110] plane where $\theta = 0 \equiv B \parallel [001]$ (as in Fig. 3(b) of main text). (b) **d**, Cross correlation of $d\rho/dB$ measured closest to $B \parallel c$ with $d\rho/dB$ measured at field tilted by θ vs θ. Also shown is the calculated R_S (eq. S-2) using the experimentally determined m^* and g[8].

modulation of the SdH oscillation by

$$R_S = \cos\left(\frac{\pi m^* g}{2\cos\theta}\right), \tag{2}$$

where m^* is the effective mass, g is the Landé g-factor and theta is the angle between the magnetic field and the high-symmetry axis, in this case the c-axis[9]. This term leads to nodes

in the SdH oscillation at specific angles and crossing the node the phase of the oscillation is reversed. Therefore, even if the precise position of the node is not observed its existence can be inferred straightforwardly.

As shown in Supplementary Figure 9(a) no node or phase reversal is observed up to angles where the amplitude is extinct at the field available in our experiment. In Supplementary Figure 9(b) we show the convolution of $d\rho(\theta)/dB$ with $d\rho/dB$ measured at approximately zero angle as a function of θ together with the expected modulation due to R_S using the quasiparticle mass measured here and the g factor measured by electron spin resonance[8]. This leads us to expect a node at approximately 55°, which is not observed.

Spin zeros have been observed in strongly correlated materials[10]. An alternative explanation for the absence of spin zeros is that m^* differs significantly between the two spin split Fermi surfaces so that at finite temperature they have different amplitudes and therefore do not produce a node. The temperature of the measurement (0.03 K) places a lower bound on m^* for the enhanced FS $> 10\ m_e$, such a large difference in m^* over 1 order in magnitude is unlikely.

Evidence for a Fermi surface transition in other Fermi surface sheets

The magnetic field dependence of the Hall coefficient, R_H, is shown in Fig. 3(e) of the main text. The Hall coefficient shows a smooth crossover between low and high magnetic fields that is temperature independent. A simple one-band interpretation of the Hall coefficient $R_H = -1/n_e$, where n is the carrier density would imply a loss of $\Delta n \approx 0.7$ electron-like carriers per formula unit. Such a large change in density is not supported by other measurements such as the resisitivty and thermodynamic measurements, we now consider a possible origin in changes in the Fermi velocity on larger FS sheets.

Supplementary Figure 10(a) shows the angular dependence of $d\rho/dB$, the overlaid thick black lines show the expected fields of B_1 and B_2 assuming they follow a 2D i.e. $1/\cos\theta$ angular dependence, while the thin vertical blue lines show the position of several magnetic field sweeps at large angles. The Fourier spectrum of these sweeps is shown in Supplementary Figure 10(b), several peaks are visible corresponding to quantum oscillations. Between 82 and 78 degrees a several peaks at $F = 0.6$, 0.9 and 1.4 kT change amplitude significantly, additionally the effective mass of the $F = 0.6$ kT is also observed to decrease by $\approx 20\%$ in

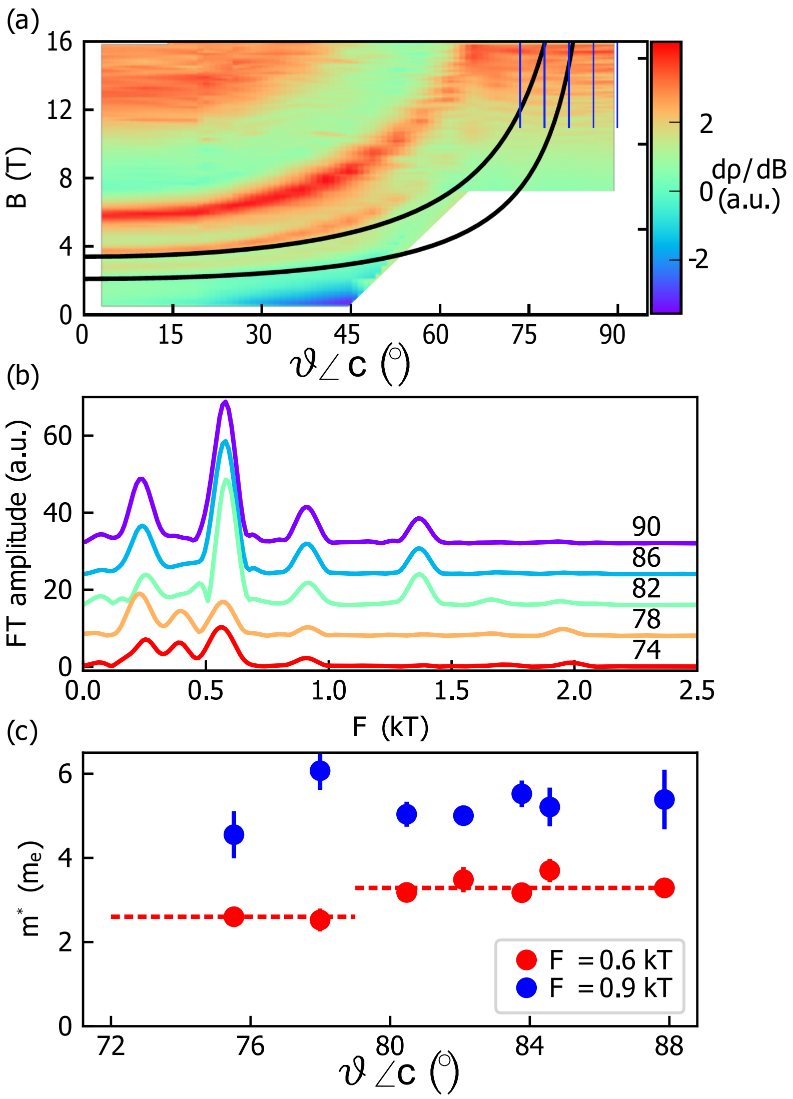

Supplementary Figure 10. (a) Angular dependence of $d\rho/dB$ at $T = 0.03\,\mathrm{K}$, black lines are guides to the eye for B_2 and $B_1/\cos\theta$. (b) Fourier transform of quantum oscillations measured in the rotation plane $[001] \rightarrow [110]$ at various θ, $T = 0.03\,\mathrm{K}$. Fourier transforms are taken over the range B = 10-16 T. (c) Angular dependence of the effective mass for the $F = 0.58$ and $0.98\,\mathrm{kT}$ orbits in the rotation plane $[001] \rightarrow [110]$.

this angular range. This angular range coincides with the speculated angular dependence of B_1 and B_2 . Therefore, we suggest that while these oscillations are not directly measurable at B_1 and B_2 for small θ, due to the Dingle factor, the Hall coefficient may undergo a crossover due to changes of the effective mass on these large FS sheets.

Shubnikov-de Haas Fourier spectrums

Supplementary Figure 11 shows Fourier spectra of $\rho_{\rm osc}$ at various temperatures, where $\rho_{\rm osc}$ is extracted from the raw resistivity as in the methods of the main text, i.e. using a local polynomial low-pass filter. Additionally we show the Fourier spectrum of $d\rho/dB$ as a dashed line, following the description of the methods section of the main text. We observe consistency between these two methods for the main peak at $F = 10.2$ T.

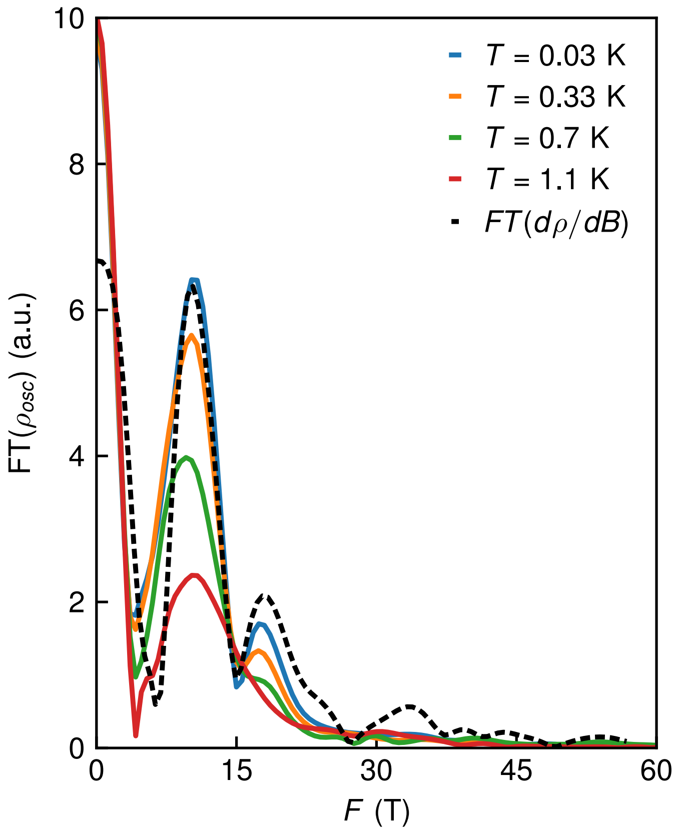

Supplementary Figure 11. Fourier spectra of the oscillatory component of the resitivity $\rho_{\rm osc}$ at several temperatures together with the spectrum of the numerical derivative of the resistivity.

SUPPLEMENTARY REFERENCES

1. Takano, S. *et al.* Site-selective ^{11}B NMR studies on $YbAlB_4$. *Journal of Physics: Conference Series* **683**, 012008 (2016).

2. Yoshimura, K. *et al.* Anomalous high-field magnetization and negative forced volume magnetostriction in $Yb_{1-x}M_xCu_2$ (M=In and Ag)– evidence for valence change in high magnetic fields. *Phys. Rev. Lett.* **60**, 851–854 (1988).
3. Terashima, T. T. *et al.* X-ray absorption spectroscopy in the heavy fermion compound α-$YbAlB_4$ at high magnetic fields. *Journal of the Physical Society of Japan* **84**, 114715 (2015).
4. Nakamura, T. *et al.* High-magnetic-field x-ray absorption and magnetic circular dichroism spectroscopy in the mixed-valent compound $YbAgCu_4$. *Journal of the Physical Society of Japan* **81**, 114702 (2012).
5. Kuga, K. *et al.* Quantum valence criticality in a correlated metal. *Science Advances* **4**, eaao3547 (2018).
6. Oura, M. *et al.* Valence fluctuating compound α-$YbAlB_4$ studied by ^{174}Yb Mössbauer spectroscopy and X-ray diffraction using synchrotron radiation. *Physica B: Condensed Matter* **536**, 162 – 164 (2018). URL `http://www.sciencedirect.com/science/article/pii/S092145261730621X`.
7. Matsumoto, Y., Kuga, K., Tomita, T., Karaki, Y. & Nakatsuji, S. Anisotropic heavy-Fermi-liquid formation in valence-fluctuating α-$YbAlB_4$. *Phys. Rev. B* **84**, 125126 (2011).
8. Holanda, L. M. *et al.* Quantum critical Kondo quasiparticles probed by ESR in β-$YbAlB_4$. *Phys. Rev. Lett.* **107**, 026402 (2011).
9. Shoenberg, D. *Magnetic oscillations in metals* (Cambridge University Press, 2009).
10. Ramshaw, B. *et al.* Angle dependence of quantum oscillations in $YBa_2Cu_3O_{6.59}$ shows free-spin behaviour of quasiparticles. *Nature Physics* **7**, 234 (2011).
